# Are U.S. cancer screening test patterns consistent with guideline recommendations with respect to the age of screening initiation?

**DOI:** 10.1186/1472-6963-9-185

**Published:** 2009-10-12

**Authors:** Srikanth Kadiyala

**Affiliations:** 1 Department of Pharmacy, Pharmaceutical Outcomes Research Policy Program, University of Washington, Seattle, Washington, USA

## Abstract

**Background:**

U.S. cancer screening guidelines communicate important information regarding the ages for which screening tests are appropriate. Little attention has been given to whether breast, colorectal and prostate cancer screening test use is responsive to guideline age information regarding the age of screening initiation.

**Methods:**

The 2006 Behavioral Risk Factor Social Survey and the 2003 National Health Interview Surveys were used to compute breast, colorectal and prostate cancer screening test rates by single year of age. Graphical and logistic regression analyses were used to compare screening rates for individuals close to and on either side of the guideline recommended screening initiation ages.

**Results:**

We identified large discrete shifts in the use of screening tests precisely at the ages where guidelines recommend that screening begin. Mammography screening in the last year increased from 22% [95% CI = 20, 25] at age 39 to 36% [95% CI = 33, 39] at age 40 and 47% [95% CI = 44, 51] at age 41. Adherence to the colorectal cancer screening guidelines within the last year increased from 18% [95% CI = 15, 22] at age 49 to 19% [95% CI = 15, 23] at age 50 and 34% [95% CI = 28, 39] at age 51. Prostate specific antigen screening in the last year increased from 28% [95% CI = 25, 31] at age 49 to 33% [95% CI = 29, 36] and 42% [95% CI = 38, 46] at ages 50 and 51. These results are robust to multivariate analyses that adjust for age, sex, income, education, marital status and health insurance status.

**Conclusion:**

The results from this study suggest that cancer screening test utilization is consistent with guideline age information regarding the age of screening initiation. Screening test and adherence rates increased by approximately 100% at the breast and colorectal cancer guideline recommended ages compared to only a 50% increase in the screening test rate for prostate cancer screening. Since information regarding the age of cancer screening initiation varies across countries, results from this study also potentially have implications for cross-country comparisons of cancer incidence and survival statistics.

## Background

Cancer screening tests are available for detecting breast, colorectal, and prostate cancers. Ideally, cancer screening tests detect cancer at an early stage when medical treatment increases the probability of survival. However, screening tests also generate false positives that may lead to the use of more invasive procedures. For breast cancer, randomized control trials and observational studies show that mammography screening reduces mortality [1,2]. A recent compendium of micro-simulation modeling studies has produced a lower bound estimate of 28% for the role of mammography screening in reducing U.S. breast cancer mortality over the 1975-2000 time period[2]. Randomized control trials also show that the fecal occult blood test (FOBT) reduces colorectal cancer mortality by 16% [3]. Randomized control trial evidence on the health benefits from the use of the PSA screening test has only recently become available [4,5]. The European trial [5] found a 20% reduction in mortality (9 year median follow up) from PSA screening but the U.S. trial [4] did not find any differences in 7 to 10 year mortality between the treatment and control groups.

Due to the complicated tradeoffs involved in the use of these screening tests, several organizations, including the American College of Physicians, American Urological Association, American College of Radiology, American Cancer Society (ACS), and the United States Preventive Services Task Force (USPSTF), have published guidelines to aid patient and physician decision-making. These guidelines generally contain recommendations on whether individuals should be screened for a specific cancer, the test to be used, the frequency with which individuals should be screened (e.g., annually, biennially, every five years), and importantly the age at which screening test use should start.

While many organizations publish cancer screening guidelines, in the United States, the guidelines published by the ACS and the USPSTF represent the vast majority of the recommendations from these other organizations. Table 1 presents the ACS and USPSTF cancer screening test guidelines for asymptomatic individuals. These guidelines were in effect during 2003 for colorectal cancer screening and 2006 for breast and prostate cancer screening--the years of the data used in the analyses in this paper [6-9]. In 2006, both the ACS and the USPSTF recommended that annual mammography screening for breast cancer begin at age 40 and that colorectal cancer screening for men and women start at age 50. Neither organization specifies the screening test that should be used to screen for colorectal cancer. In the case of prostate cancer, the recommendations from the two organizations differ. The ACS recommends that physicians offer the prostate specific antigen (PSA) test annually to individuals starting at age 50, while the USPSTF finds insufficient evidence to determine whether individuals should receive the PSA test. In this instance, the ACS favors shared decision-making between the physician and patient with respect to PSA screening [10].

**Table 1:** Cancer Screening Test Guidelines for Asymptomatic Populations (Effective 2003 for Colorectal Cancer and 2006 for Breast and Prostate Cancers)

**Panel A: Colorectal Cancer Screening**	American Cancer Society (2001):	- Annual fecal occult blood test ages 50+
		- Or sigmoidoscopy every 5 years starting at age 50
		- Or annual FOBT and flex. Sig. Every 5 years starting at 50
		- Colonoscopy every 10 years starting at age 50
		- Double contrast barium enema every 5 years starting at 50
	United States Preventive Services Task Force (2001-2004):	- Screening recommended ages 50+
		- Annual fecal occult blood test and or periodic sigmoidoscopy

**Panel B: Prostate Cancer Screening**	American Cancer Society (2004):	- Prostate specific antigen test offered annually to men ages 50+ with 10+ years life expectancy
	United States Preventive Services Task Force (2005):	- Insufficient evidence to recommend or not recommend the test

**Panel C: Breast Cancer Screening**	American Cancer Society (2004):	- Annual mammography ages 40+
	United States Preventive Services Task Force (2005):	- Mammography every 1-2 years ages 40+

Source: ACS Guidelines, Smith et al. (2004). ^a^USPSTF 2002 (For Colorectal Cancer Screening). ^b^USPSTF 2002 (For Prostate Cancer Screening) ^c^USPSTF 2002 (For Breast Cancer Screening)

An extensive academic literature has examined the use of screening tests in the ages recommended by the guidelines [11-26]. A recent paper by Smith et al. (2007) [26] uses the 2004 Behavioral Risk Factor Surveillance System (BRFSS) data to evaluate screening rates. They find that 58% of women ages 50-64 received a mammogram within the previous year, 52% of women and men ages 50-64 received either a home FOBT within the past year year or a sigmoidoscopy or a colonoscopy within the past 5 years, and 54% of men ages 50-64 received a PSA test within the previous year.

We extend this line of research by explicitly evaluating whether breast, colorectal, and prostate cancer screening test rates are consistent with guideline age information. By recommending an age to start screening, guidelines imply that screening is more valuable for individuals of a certain age. Given that physicians report adhering to cancer screening guidelines [13,27] and state health insurance mandates based on the ACS and USPSTF guidelines reduce the price of screening tests [28], a key yet unexplored question in this literature then becomes whether screening test behavior is responsive to guideline age recommendations. This question of age appropriate screening is studied by evaluating breast, colorectal and prostate cancer screening test rates for individuals close to and on either side of the guideline recommended ages. A simultaneous evaluation of the breast, colorectal and prostate cancer screening will also provide insight into whether physician and patient response is related to differences in the ACS and USPSTF recommendations. As noted above, for breast and colorectal cancer screening there is agreement across the ACS and USPSTF guidelines that asymptomatic individuals should receive the test. In the case of the PSA test, there is no agreement across the guidelines that the test should be utilized, and the ACS recommendation is weaker in that it only requires an offer of the test.

## Methods

### Data Source

The following analyses were conducted using data from the 2006 BRFSS and the 2003 National Health Interview Survey (NHIS) sample adult file [29,30]. Both the BRFSS and the NHIS collect information on demographics, health, and health care use of the non-institutionalized U.S. population. Both are annual surveys, use stratified sampling methods for data collection and are designed to provide estimates that are representative of the U.S. non-institutionalized population. Individuals of all ages are eligible to be sampled in the NHIS but the BRFSS primarily samples individuals 18 years and older. Most importantly, the surveys include information on adult cancer screening test use and the timing of the last cancer screening test, i.e., whether the screening occurred within the past year, two years, or further back. More documentation about the design of the surveys is available at the BRFSS and NHIS data sites [29,30].

Breast cancer screening test data for women aged 30-70 and prostate cancer screening test data for men aged 40-70 is taken from the 2006 BRFSS. The colorectal cancer screening test data for men and women aged 40-70 is from the 2003 NHIS. The NHIS data is analyzed in conjunction with the BRFSS data because the BRFSS only collects colorectal cancer screening test information for men and women ages 50 and older. Reporting for screening test use is extremely high in these surveys. In the BRFSS, 97% of women aged 30-70 (N = 153,606) and 92% of men aged 40-70 (N = 75,285) report their mammography and PSA screening test behavior. In the NHIS, 95% of adults aged 40-70 (N = 13,991) years report on their colorectal cancer screening test behavior.

### Outcomes

For breast cancer we calculated the proportion of women who received a mammogram in the past 1 and 2 years. For colorectal cancer the USPSTF and the ACS specify annual FOBT use and that the FOBT sample be collected at home. Guidelines also recommend either sigmoidoscopy use once every 5 years or colonoscopy use once every 10 years. Neither guideline takes a stance on which colorectal screening test to perform. Consequently, we consider someone as adherent with the colorectal cancer screening guidelines if they received either a home FOBT in the last year, a sigmoidoscopy in the last 5 years or a colonoscopy in the last 10 years. We also evaluated the share of individuals who received either a home FOBT within the last 2 years, a sigmoidoscopy within the last 5 years or a colonoscopy within the last 10 years. For prostate cancer we calculated the share of men who received a PSA screening test in the past 1 and 2 years.

### Analytic Strategy

In evaluating whether screening test use is consistent with guideline age information, we examined screening rates at the age thresholds where guidelines recommend that screening begin. If screening rates are consistent with guideline age information then one would expect to see a large, discrete increase in screening precisely at the guideline recommended screening initiation ages. Within this framework, even if screening rates at the recommended age thresholds are high, a finding that screening rates right below the recommended ages are equivalent would suggest that screening utilization is not consistent with guideline age information. These results would suggest the simpler explanation that all individuals receive the same amount of screening and that age information contained in guidelines is not related to individual or physician decision-making. Similarly, even if screening rates at the recommended ages are low, screening patterns can still be consistent with guideline age information. For example, even if the colorectal cancer-screening rate is only 30% at the recommended ages, it is possible for screening to be responsive to guideline age information if the data show that the colorectal screening rate just below the recommended ages is only 15%. This change in screening precisely at the guideline recommended ages would imply that colorectal cancer screening use at the recommended age threshold increased by 100%.

In the following analyses, we first provide graphical evidence with respect to the relationship between age and breast, colorectal and prostate cancer screening test rates. We then formalize the analytic strategy in the regression framework by fitting the following logistic regression model, Pr [*Y *= 1] = *B*_1_(*age*) +*B*_2 _*(guideline) *where the dependent variable is utilization of breast, colorectal and prostate cancer screening tests.

In this specification, "age" is a linear variable, while the "guideline" variable is a dummy variable that assumes a value of 1 if the individual is within the guideline recommended ages for that screening test and zero otherwise. The B_1 _coefficient estimates how screening relates to age, while the B_2 _coefficient estimates the change in screening from being at an age where screening is recommended. This analysis allows us to isolate the shift in the screening rate that occurs for individuals within the guideline recommended ages, relative to individuals outside of the guideline recommended ages, from the normal increase in screening test use that occurs with age [31].

In the regression analysis, to adjust for potential confounding by socioeconomic and demographic variables related to screening [15,24,32-34], we also estimated regression models that include income, health insurance, education, race and marital status. In these regressions, race is defined as white or non-white. An individual is defined as having health insurance if they are covered by a private or public health insurance plan. Education is grouped into four categories: less than high school, high school graduate, some college, and college graduate or higher (omitted regression category is "less than high school"). We also adjust for marital status by using the following categories: married/partnered, divorced or separated, widowed and never married (omitted regression category is married/partnered). We group the BRFSS income data in to 4 groups: < $15,000; $15,000 to $24,999; $25,000 to 434,999; and > $35,000. In the NHIS data, we match the BRFSS income categorizations, but due to differences in income reporting between the NHIS and the BRFSS we add two other dummy variables: one variable for individuals who report their annual household income as less than $20,000, and one variable for individuals who report their annual household income as greater than $20,000.

Finally, the BRFSS and the NHIS surveys use stratified sampling methods to collect survey information and the results reported in this study take the survey weights and complex sampling frames into account. All of the following analyses were conducted in Stata Version 10.

## Results

### Graphical Analysis

#### Breast Cancer

**Figure 1:**
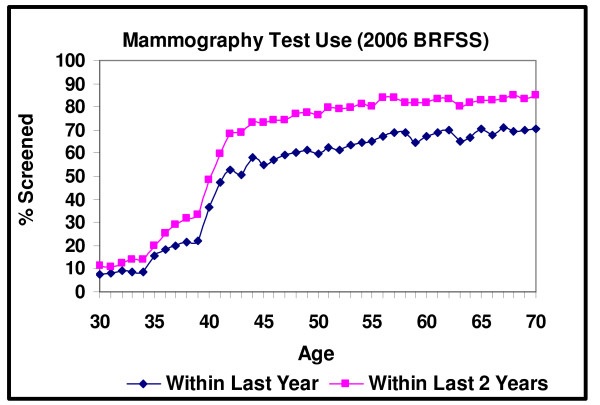
**Mammography Test Use within the Last 1 and 2 Years**.

Figure 1 is a plot, using the 2006 BRFSS data, of the share of women who received a mammogram in the past year and the past two years by age. In Figure 1, a large discrete shift in the percentage of women of being screened occurs at exactly the age that guidelines recommend that screening begin. The share of 39 year old women screened in the last year is 22%, and this figure rises to 36% (p < .001) for a woman who is 40 years old and 47% (p < .001) for a woman who is 41 years old. Using the past two years as a screening measure shows similar results. Screening rises from 34% at age 39 to 48% (p < .001) at age 40 to 60% (p < .001) at age 41. One issue to note regarding the difference between the age 40 and age 41 screening rates is that not everyone who is 40 years old has been at the recommended screening age for an equivalent amount of time. This is because the screening measure used in the BRFSS is "screened in the past year", which means that women who turned 40 years of age close to the BRFSS interview date have spent little time in the recommended screening age. Consequently, the screening test rate for a 40 year-old understates the potential response to an individual's age.

#### Colorectal Cancer

**Figure 2:**
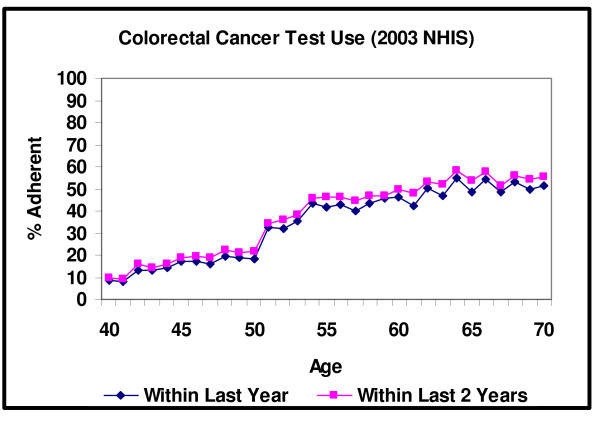
**Colorectal Cancer Test Use within the Last 1 and 2 years**. Note: An individual is adherent with the colorectal cancer guidelines within the last year if they received either an FOBT within the last 1 year, a sigmoidoscopy within the last 5 years or a colonoscopy within the last 10 years. Adherence within the last 2 years is defined in a similar manner except individuals may receive an FOBT within either the last 1 or 2 years.

Figure 2 plots the share of men and women who received either a home FOBT within the last year, a sigmoidoscopy within the last 5 years or a colonoscopy within the last 10 years. As with mammography, there is a large increase in colorectal cancer screening that occurs precisely at the age specified by guidelines. Adherence to the guideline recommendations within the last year rises from 18% at age 49 to 19% (p = .865) at age 50 and 33% (p < .001) at age 51. Similar to the adherence in the last year measure, there is an increase in the share of individuals who are adherent within the past 2 years from 21% at age 49 to 22% (p = .847) at age 50 and 34% (p < .001) at age 51. We also analyzed the colorectal cancer screening data separately by sex but did not find any statistically significant differences between the two groups with respect to changes in screening near age 50. Overall, the absolute changes in colorectal screening adherence at the precise thresholds are smaller when compared to mammography, but the relative changes are similar. In both cases the adherence measure increases by approximately 100% within two years of the recommended age thresholds.

### Prostate Cancer

**Figure 3:**
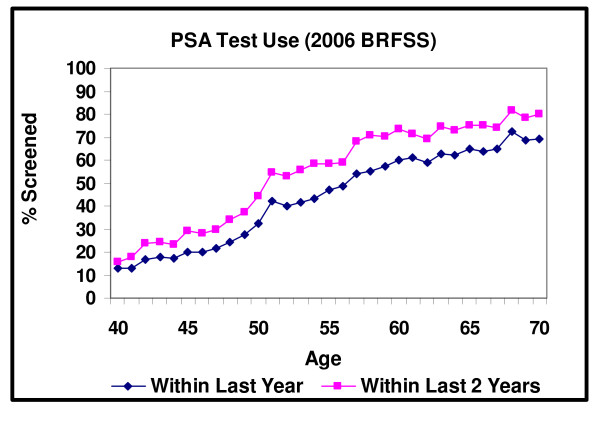
**PSA Test Use within the Last 1 and 2 Years**.

Figure 3 presents data from the 2006 BRFSS on PSA screening test use within the last year and two years by age. The share of men screened within the past year rises by 5 percentage points, from 28% at age 49 to 33% (p = .035) at age 50, and by 14 percentage points (p < .001) from age 49 to age 51. The share of men screened within the last two years also increases by a significant amount, 17 percentage points (p < .001) from age 49 to age 51. Previous work [14,17] has shown that PSA screening is performed far more frequently than colorectal cancer screening. While prostate cancer screening use is greater than colorectal cancer screening use for a given age, the prostate cancer screening changes at the guideline recommended ages are smaller. PSA screening test use only increases by 50% at the guideline recommended ages as compared to the approximately 100% increases in the use of the breast and colorectal cancer screening measures.

### Regression Analysis

Table 2 presents marginal effects from logistic regression models for breast and prostate cancer screening test use within the last year and colorectal cancer screening guideline adherence within the last year. For these analyses, to get precise guideline estimates, the sample for each regression was restricted to individuals within 10 years above and below the age that guidelines recommend that screening start. For breast cancer (column 1), after adjusting for the underlying trend of increased screening with age, being in the guideline recommended ages increases the probability of receiving a mammogram in the last year by 21% points (p < .001) relative to individuals not in the guideline recommended ages. Similarly, for colorectal and prostate cancers (columns 3 and 5), being in the guideline recommended ages increases colorectal cancer guideline adherence and PSA test use in the last year by approximately 6.0% points (p = .009) and 6.8% points (p < .001). For each of the screening types, the coefficient on the linear age variable is also positive and statistically significant (p < .001). Most importantly, the significant shifts identified in the graphical analyses still remain for all the screening tests even after adjusting for age trends.

**Table 2:** Logistic Regression Models for Breast, Prostate and Colorectal Cancer Test Use

	**BREAST CANCER** **(Mammography Use Within ** **the Last Year)**		**COLORECTAL CANCER** **(Adherent to Guidelines Within** **the Last Year)**		**PROSTATE CANCER** **(PSA Test Use Within ** **the Last Year)**	
**Variables**	**Unadjusted Marginal Effects** **(95% CI)**	**Adjusted** **Marginal Effects** **(95% CI)**	**Unadjusted Marginal Effects** **(95% CI)**	**Adjusted** **Marginal Effects** **(95% CI)**	**Unadjusted Marginal Effects** **(95% CI)**	**Adjusted** **Marginal Effects** **(95% CI)**

**Age**	0.022(.019,.024)**	0.022(.020,.025)**	0.017(.013,.020)**	0.017(.013,.020)**	0.021(.018,.023)**	0.021(.018,.023)**

**Guideline**	0.211(0.184,.238)**	0.212(.185,.238)**	0.060(.016,.104)**	0.056(.014,.100)**	0.068(.037,.100)**	0.067(.035,.099)**

**Rac**e - White+	-		-		-	
Non-White		0.073(.055,.092)**		-0.015(-.040,.010)		0.061(.021,.071)**

**Health Insurance**	-	0.162(.142,.183)**		0.141(.118,.164)**		0.157(.132,.182)**

**Income**	-		-		-	
Inc. < $15000^+^						
Inc. $15000						
-24999		0.047(.007,.088)*		-0.020(-.068,.029)		- 0.021(-.073,.029)
Inc. $25000						
34999		0.018(-.023,.059)		-0.032(-.074,.011)		- 0.016(-.067,.035)
Inc. $35,000 plus		0.061(.028,.094)**		0.016(-.022,.054)		0.025(-.021,.071)
Inc. < $20000		-		0.006(-.039,.051)		
Inc. > $20000				-0.00(-.060,.059)		

Marital Status			-		-	
Married/partnered^+^						
Divorced						
Widowed		-0.007(-.028,.014)		-0.001(-.025,.023)		-0.035(-.060,-.009)
Never married		-0.05(-.096,.0002)		0.029(-.025,.084)		0.051(-.009,.110)
		-0.012(-.038,.014)		-0.008(-.040,.025)		-0.040(-.07, 01)**

**Education**	-		-		-	
< High school^+^						
High school grad.		-0.006(-.046,.033)		0.093(.053,.134)**		0.055(.006,.103)*
Some college		-0.002(-.041,.038)		0.111(.072,.150)**		0.095(.043,.148)**
College grad or higher		0.018(-.022,.058)		0.148(.107,.190)**		0.108(.058,.158)**

**Sample Size**	69547	69547	10127	10127	50028	50028

Source: Mammography and PSA data are from the 2006 BRFSS, Colorectal cancer test data are from the 2003 NHIS Adult Subsample, ^+^Reference Category, *P < 0.05, ** P < 0.01.

Notes: Estimates are marginal effects from logistic regression models. For breast cancer, the regression sample is all women ages 30-50. For colorectal cancer the regression sample is all men and women ages 40-60. For prostate cancer the regression sample is all men ages 40-60. In each of the regressions, the "guideline'' variable assumes a value of 1 if the individual is within the guideline recommended age, and zero otherwise. Someone is defined as being adherent to the colorectal cancer screening guideline if they received either a FOBT within the past year, a sigmoidoscopy within the last 5 years or a colonoscopy within the last 10 years. BRFSS and NHIS income coding is different. The differences are described in the text.

Columns II, IV and VI in Table 2 adjust for potentially important confounders such as income, health insurance, education, race and marital status. The coefficients identified for individuals in the guideline recommended ages are robust to the inclusion of demographic and socioeconomic controls, as the guideline estimates from both regression model specifications for all the screening measures are almost identical (row 2, Table 2). This implies that differences in demographic and socio-economic variables, for individuals within and outside the guideline recommended ages, cannot account for the observed screening changes.

One potential concern with the BRFSS and NHIS results is that the cancer screening guideline recommendations are specifically for screening asymptomatic individuals and not meant for diagnostic purposes. In the BRFSS data it is not possible to distinguish between diagnostic use and routine screening, but this distinction is possible in the NHIS data. In the NHIS data, individuals are asked whether their most recent colorectal cancer test was performed as part of a routine screen (or routine physical exam) or for diagnostic reasons due to a specific problem.

Analysis of the NHIS reason for test data supports the claim that the change in colorectal cancer test adherence at age 50 is primarily driven by an increase in asymptomatic screening. Asymptomatic colorectal cancer screening adherence rises from 11% at age 49 to 12% (p = .354) at age 50 and 21% (p < .001) at age 51. Estimating a logistic regression model, with asymptomatic colorectal screening adherence in the past year as the dependent variable, and the other covariates from Table 2 shows that the probability of asymptomatic screening adherence increases by 5.9% (p < .001) points for individuals in the guideline recommended ages. This estimate of the change in asymptomatic screening adherence from being in the guideline recommended age group is nearly identical to the guideline estimate presented in column 4 of Table 2.

The result that most of the change in colorectal cancer test adherence at the guideline recommended age threshold is due to a change in asymptomatic colorectal cancer test use is perhaps not surprising. This is because there are no theoretical or empirical reasons, either from biology or epidemiology, to expect that the underlying incidences of breast, colorectal and prostate cancers increase discretely at the guideline recommended age thresholds. Thus one should not have expected a discrete increase in non-asymptomatic colorectal diagnostic test use near age 50. These asymptomatic colorectal cancer test results suggest that the mammography and PSA guideline estimates identified in Table 2 primarily capture changes in asymptomatic mammography and PSA test use.

## Discussion

Identification of the extent to which screening test patterns are consistent with guideline age information is important since guideline age thresholds convey information regarding the tradeoffs between screening benefits and harms. The empirical results from the analyses of the BRFSS and the NHIS data suggest that cancer screening test behavior by physicians and patients is influenced by age recommendations, although there are significant differences in the magnitudes across the cancer types. Mammography screening increases by 25 percentage points from age 39 to age 41; this is more than a 100% increase relative to the age 39 baseline screening rate. For colorectal cancer there is a relative 90% increase in adherence while PSA screening only increases by 50% at the guideline recommended ages. The larger relative increase in mammography and colorectal cancer screening test use, when compared to PSA screening test use, is consistent with guideline recommendations for the use of these tests being much stronger than the guideline recommendations for the use of the PSA test.

An important contribution of this research is the idea that analysis of cancer screening test rates on either side of the guideline recommended age thresholds is necessary to understand the full impact of guideline age recommendations. Since screening is not recommended for individuals who are younger than the guideline recommended age thresholds, the test rates presented in this paper for individuals in these groups represent *upper bound *measures for the overuse of these tests. Considering the results from this perspective, the PSA test is potentially the most overused test, as 19% of men aged 40-49 received the test in the past year. Mammography use in 30-39 year old women is also substantial as 14% of women received this test in the last year. Similarly, 14% of men and women aged 40-49 also received either a FOBT in the past year, a sigmoidoscopy in the past 5 years or colonoscopy in the last 10 years. For colorectal cancer, only taking into account test use for asymptomatic reasons reduces the 14% estimate of overuse to 8%.

It is necessary to note that even after crudely separating out asymptomatic screening from total test use, these calculations are likely to be strict measures of breast, prostate and colorectal cancer screening overuse. This is because guideline age recommendations are based primarily on considerations regarding the tradeoffs between population health benefits and population health costs. Guidelines do not take into account individual preferences for the use of screening tests (such as risk-aversion and time preference) [35] and do not take into account an individual's demand for health status information that is unlikely to affect their long run health outcomes. Just as research on screening has attempted to understand the reasons behind low screening rates in the guideline recommended ages [36], future research should also consider exploring the reasons for the use of screening tests in the ages where screening is not recommended.

Although this research has identified the extent to which screening is consistent with guideline age recommendations, the mechanisms behind the observed increases in screening are not well understood. Prior work has shown that physician offer is an important predictor of screening utilization [36] and it is likely that some combination of an increase in physician offer of screening at the guideline recommended ages and an increase in an individual's demand for screening at the guideline recommended ages explains the results identified in this study. Physician offer and patient demand in turn are likely affected by physician and individual knowledge regarding guidelines [13,27], responses to the coverage of screening tests at the guideline recommended ages by insurance [28], psychosocial factors and U.S. medical malpractice laws. The results from this study also potentially have implications for international comparisons of cancer incidence and survival statistics. Compared with the United States, guidelines for several OECD countries (for example Canada and U.K) have differing recommendations with respect to the timing of breast and prostate cancer screening [37,38]. Most notably, mammography screening is recommended to begin at or near age 50, the PSA test is not recommended for asymptomatic individuals and guidelines also recommend stopping ages for screening. If screening rates in other OECD countries are consistent with their own screening recommendations, then understanding cross-country differences in the timing of screening initiation and termination between the U.S. and other countries can potentially aid in interpreting comparisons of cancer incidence and survival statistics.

There are several limitations to the results identified in this study. The most significant limitation is the inability to distinguish between diagnostic testing and asymptomatic screening in the case of breast and prostate cancers. Such differentiation is possible for colorectal cancer screening in the NHIS data but not possible for prostate and breast cancer screening in the BRFSS data. A second limitation is that both the BRFSS and the NHIS use self report as the primary means of data collection and it is possible that individuals made errors in remembering the time frame in which they were screened or errors in whether or not they received the test [39]. This is potentially important in the case of the PSA test, a test that individuals might have trouble differentiating from a blood test. Although there is no direct way to evaluate the magnitude of these errors, it is important to note that errors in memory and confusion would have to arise precisely at the guideline recommended ages to explain the results identified in this paper. We are not aware of any evidence that suggests that errors are magnified at the guideline recommended ages.

## Conclusion

This study has provided new evidence on whether breast, colorectal and prostate cancer screening rates are responsive to guideline recommendations with respect to the age of screening initiation. The results indicate that there are substantial increases in breast, colorectal and prostate cancer screening precisely at the guideline recommended ages, although little is currently known regarding the mechanisms behind these observed increases. This research also provides a crude upper bound estimate for the amount of overuse of the colorectal cancer screening tests in the ages where asymptomatic screenings are not recommended. Future research should consider the reasons why asymptomatic individuals that are outside of the guideline recommended age thresholds receive screening tests. The question of age appropriate screening has risen again recently as the USPSTF guidelines now recommend that males 75 and older not be screened with the PSA test since the screening harms outweigh the screening benefits for this age group [40]. Future research should also evaluate the extent to which PSA screening patterns reflect this new USPSTF age recommendation. Finally, cancer screening guideline information varies across countries. Understanding how this variation in guideline information affects country screening patterns will likely aid in the interpretation of cross-country comparisons of cancer incidence and mortality statistics.

## Abbreviations

ACS: American Cancer Society; BRFSS: Behavioral Risk Factor Social Survey; NHIS: National Health Interview Survey; PSA: Prostate Specific Antigen; OECD: Organization for Economic Co-Operation and Development; USPSTF: United States Preventive Services Task Force.

## Competing interests

The author declares that they have no competing interests.

## Pre-publication history

The pre-publication history for this paper can be accessed here:


